# The *Saccharomyces cerevisiae* poly(A) binding protein Pab1 as a target for eliciting stress tolerant phenotypes

**DOI:** 10.1038/srep18318

**Published:** 2015-12-14

**Authors:** Francesca Martani, Francesca Marano, Stefano Bertacchi, Danilo Porro, Paola Branduardi

**Affiliations:** 1 Department of Biotechnology and Biosciences, University of Milano-Bicocca, Milano, 20126, Italy; 2 SYSBIO – Centre of Systems Biology, Milano and Roma, Italy

## Abstract

When exploited as cell factories, *Saccharomyces cerevisiae* cells are exposed to harsh environmental stresses impairing titer, yield and productivity of the fermentative processes. The development of robust strains therefore represents a pivotal challenge for the implementation of cost-effective bioprocesses. Altering master regulators of general cellular rewiring represents a possible strategy to evoke shaded potential that may accomplish the desirable features. The poly(A) binding protein Pab1, as stress granules component, was here selected as the target for obtaining widespread alterations in mRNA metabolism, resulting in stress tolerant phenotypes. Firstly, we demonstrated that the modulation of Pab1 levels improves robustness against different stressors. Secondly, the mutagenesis of *PAB1* and the application of a specific screening protocol on acetic acid enriched medium allowed the isolation of the further ameliorated mutant *pab1* A60-9. These findings pave the way for a novel approach to unlock industrially promising phenotypes through the modulation of a post-transcriptional regulatory element.

The yeast *Saccharomyces cerevisiae* is widely employed as cell factory for the production of several industrial products, such as fine and bulk chemicals, pharmaceutics, and biofuels^1^,^2^. During industrial fermentations, yeasts meet multiple stresses associated with the operative parameters of the process that, together with the final product toxicity, contribute to slowing down cell metabolism and growth^3^,^4^. Moreover, and in particular during second-generation production processes, the presence of inhibitory compounds negatively affect cell performance and the consequent titer, yield and productivity of the fermentative process^5^. The evolution of robust cell factories is therefore desirable to attain higher production and productivity, which are essential prerequisites to achieve viable and competitive bioprocesses^6^. Several approaches have been applied to improve *S. cerevisiae* robustness and to reduce the negative effects of both inhibitors and stressful conditions imposed by industrial fermentations. In literature there are several examples of strains with increased stress tolerance obtained by genetic engineering through the deletion or overexpression of single genes involved in a particular stress response^7^,^8^,^9^,^10^,^11^. However, the evolution of a robust phenotype is hardly obtainable through the alteration of a molecular element with finite action, since the stress response is a complex trait resulting from coordinated changes at molecular and cellular level^12^. Robust industrial strains were therefore developed by genome-scale engineering, using techniques such as mutagenesis, protoplast fusion, breeding, genome shuffling and directed evolution, which rely on the creation of diversity and the selection of the desired phenotype^13^,^14^,^15^. As alternative, the modulation of “hub elements”, responsible for cellular reorganization, can be explored.

In the last years, the remodeling of the transcriptome by global transcription machinery engineering (gTME) has been applied as a powerful strategy for the obtainment of complex phenotypes, including stress tolerance improvement^16^. In response to stress, the fine tuning of gene expression plays indeed a key role in the activation of molecular mechanisms required for cellular adaptation to new environments^17^. Although transcription shapes the adaptive response to stress, the mechanisms regulating the fate of newly synthesized mRNAs are crucial for tuning the final effect of eukaryotic gene expression^18^,^19^. During their lifetime, cytosolic mRNAs are dynamically bound to proteins in different messenger ribonucleoprotein (mRNP) complexes, which regulate their translation, turnover and subcellular localization^20^. Translating mRNAs are usually trapped into polysomes, while non-translating mRNAs can accumulate in mRNA-protein complexes named processing bodies (P-bodies) and stress granules (SG)^18^,^21^,^22^. These RNA granules exert a key role in the modulation of post-transcriptional regulation of gene expression, particularly during the cellular stress response^23^.

P-bodies contain proteins associated with the mRNA decay machinery and so mainly mRNAs addressed to be degraded^22^. Accordingly, P-bodies can be present in both unstressed and stressed cells, but in the latter, characterized by the inhibition of translation, their formation is exacerbated^24^. In contrast, SG are only present in the cytoplasm of stressed cells and differ from P-bodies in protein composition and function^22^. SG usually contain mRNAs bound with translation initiation factors, 40S ribosomal subunits and the poly(A) binding protein, and are therefore believed to represent sites of both mRNA protection from decay and translation reinitiation, allowing a rapid resume of translation throughout their dissolution^22^. In *S. cerevisiae*, P-bodies have been demonstrated to promote SG assembly, suggesting the existence of a mRNA cycle in which mRNPs are exchanged between these cytoplasmic granules^24^,^25^.

Yeast SG have been studied for their assembly and composition mainly under glucose deprivation or severe heat shock^25^,^26^,^27^,^28^, but they were also observed in the presence of other type of stress^29^,^30^. Although SG protein composition differs depending on the stressful condition, the yeast main poly(A) binding protein Pab1 represents a specific component, commonly used as a SG marker in its GFP-tagged form^22^,^26^. Moreover, Pab1 regulates many other aspects of the mRNA metabolism through its simultaneous binding to the mRNAs poly(A) tails and interaction with several proteins involved in mRNA biogenesis and decay^31^,^32^,^33^.

Therefore, SG emerge as prominent targets for the development of complex phenotypes, and we have consequently identified Pab1 as promising candidate to be manipulated for the development of robust yeasts. In this work, we demonstrated that the overexpression of *PAB1* on a centromeric plasmid increases *S. cerevisiae* resistance to different stresses commonly occurring during industrial fermentations. This robust phenotype was further improved through the selection of a mutant version of Pab1, under selective pressure for acetic acid tolerance. In both cases, an effect on SG morphology was also observed.

## Results

### Effect of the overexpression of *PAB1* on *S. cerevisiae* growth under stressful conditions

*S. cerevisiae* cells in which *PAB1* was overexpressed under the control of the strong constitutive TEF promoter were unviable, consistently with the reported evidences that the galactose-inducible *PAB1* overexpression causes severe growth inhibition^28^. High expression levels of this gene are therefore not tolerated by yeast cells.

**Figure 1:**
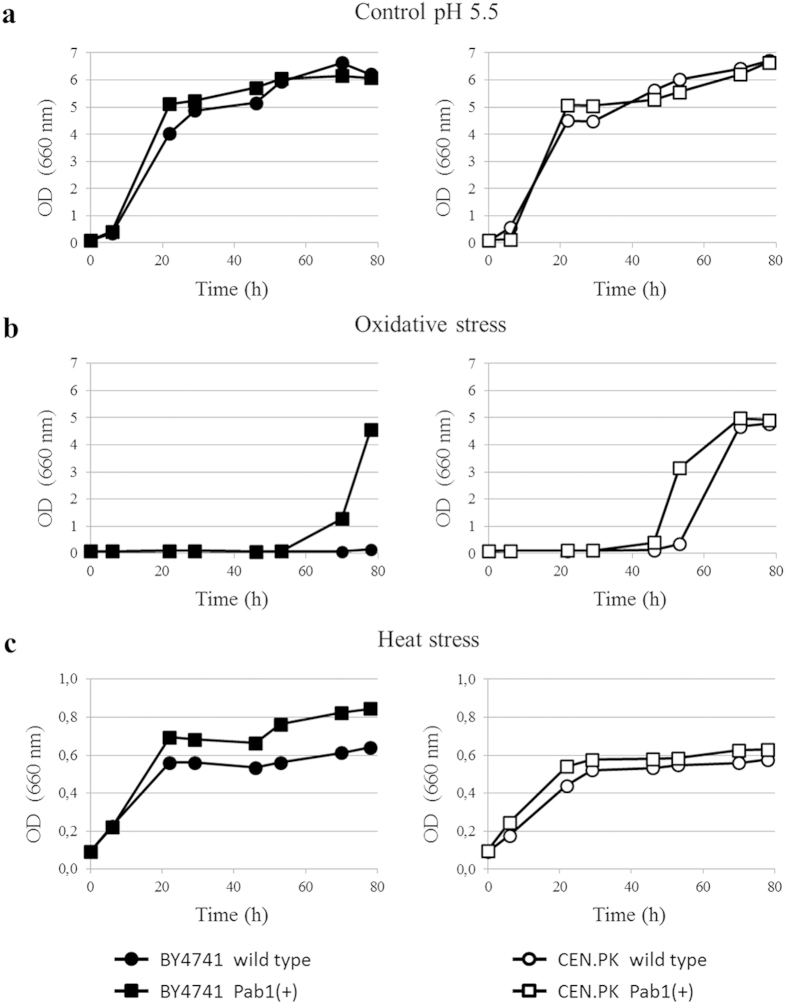
Effect of the overexpression of *PAB1* on *S. cerevisiae* stress tolerance. Wild type and Pab1(+) strains of BY4741 and CEN.PK backgrounds were grown in minimal medium at pH 5.5 (**a**) in the absence of stress, (**b**) in the presence of 4 mM H_2_O_2_, and (**c**) at 42 °C. Note the different scale of the ordinate axis of growth curves performed at 42 °C. The results of one representative experiment of three are shown.

**Figure 2:**
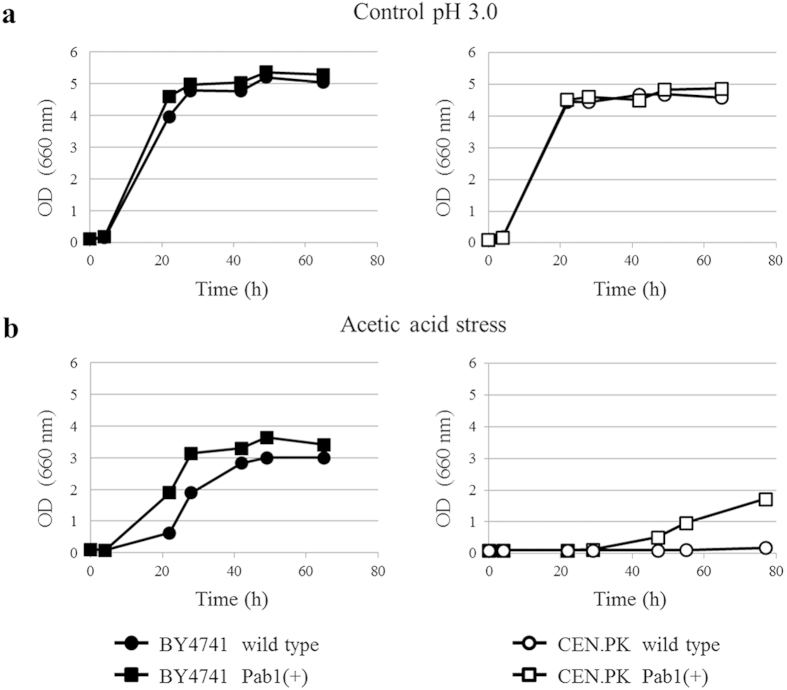
Effect of the overexpression of *PAB1* on *S. cerevisiae* tolerance to acetic acid. Wild type and Pab1(+) strains of BY4741 and CEN.PK backgrounds were grown in minimal medium at pH 3.0 (**a**) in the absence and (**b**) in the presence of acetic acid. The results of one representative experiment of three are shown.

Consequently, we assessed *in vivo* the impact of the overexpression of *PAB1* at low gene dosage by analyzing cell growth of *S. cerevisiae* strains transformed with the centromeric plasmid YCplac33 harboring the wild type ORF of *PAB1* under the control of a part of its endogenous promoter (500 bp), identified with the Promoter Database of *S. cerevisiae* (http://rulai.cshl.edu/SCPD). This portion of the promoter was demonstrated to be sufficient for *PAB1* expression by western blot analysis of Pab1-GFP fusion protein and by fluorescent microscopy (data not shown). The prototrophic strains containing the plasmid YCplac33*PAB1* were named Pab1(+) (Table S1). To collect data in different genetic backgrounds, BY4741 (Figs 1 and 2, left panels) and CEN.PK (Figs 1 and 2, right panels) yeast strains were used and analyzed in parallel. Densitometry of western blot bands revealed an increase of about 60% of Pab1 expression in Pab1(+) compared to control cells (see supplementary Fig. S1 online).

In minimal medium at pH 5.5 cell growth was not significantly affected by the increased expression of *PAB1* in both yeast strains (Fig. 1a). Successively, growth kinetics of wild type and Pab1(+) strains in the presence of oxidative (4 mM H_2_O_2_; Fig. 1b) and heat (42 °C; Fig. 1c) stress were compared. As shown in Fig. 1, Pab1(+) cells were in general more tolerant to the stressful conditions compared to wild type cells in both genetic backgrounds. The different growth kinetics showed by BY4741 and CEN.PK in the presence of the stressors are most likely ascribed to the genotypic and phenotypic differences of the parental strains from which they were obtained^34^. Cell growth was also analyzed in the presence of acetic acid, one of the most abundant and toxic compounds released during pre-treatment of lignocellulose^4^,^35^. In minimal medium at pH 3.0 (here used as a control condition), the growth was comparable for both wild type and Pab1(+) strains (Fig. 2a). However, Pab1(+) cells exposed to acetic acid showed a growth advantage compared to wild type cells (Fig. 2b), confirming the robust phenotype towards stressful conditions observed in Fig. 1. All together, these data show that *PAB1* overexpression at low gene dosage is not lethal and, indeed, increases yeast stress tolerance.

### The overexpression of *PAB1* alters SG morphology during heat stress

**Figure 3:**
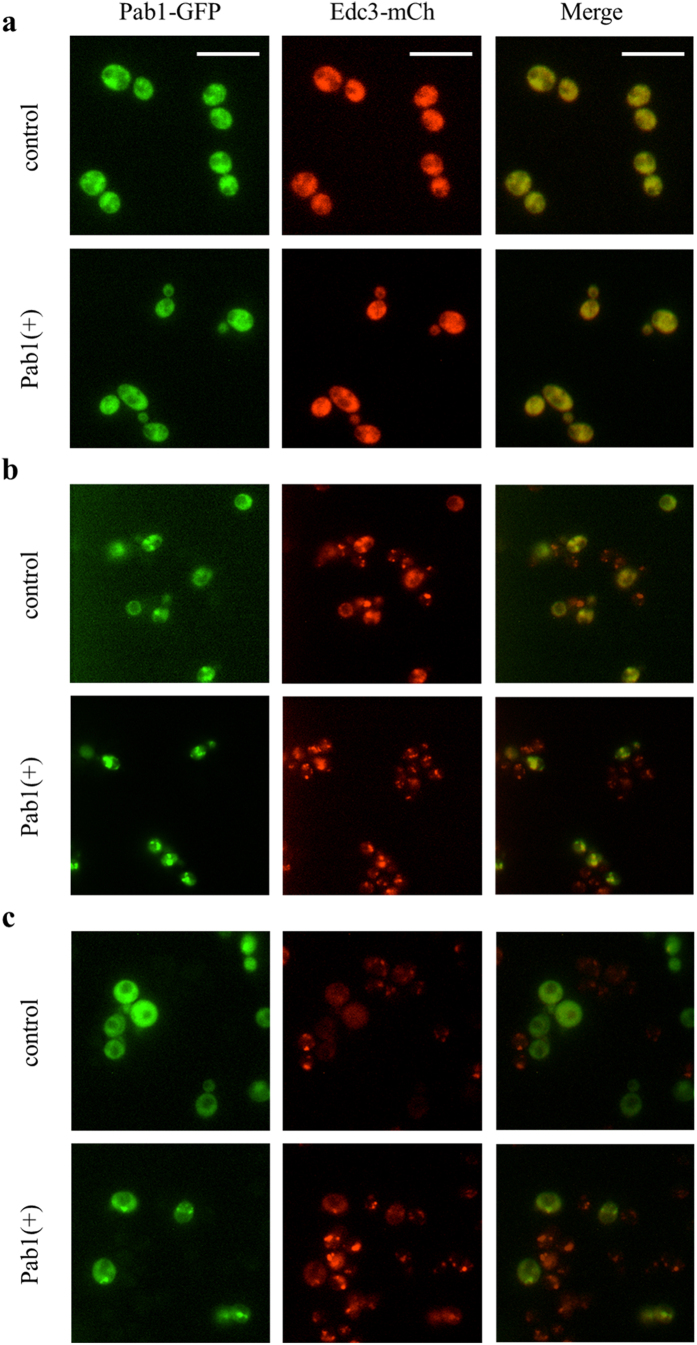
Effect of the overexpression of *PAB1* on *S. cerevisiae* SG and P-bodies morphology. Pab1 and Edc3 associated fluorescence of wild type (control) and Pab1(+) strains was analyzed (**a**) in the absence of stress, (**b**) after incubation at 46 °C for 2 h, and (**c**) after 20 min of recovery at 30 °C. Scale bar, 10 μm.

The effect of the elevated expression of *PAB1* on SG and P-bodies assembly was then analyzed. To compare SG between wild type and Pab1(+) strains, we used a strain containing the chromosomally integrated GFP-tagged version of Pab1 as the control, and the strain PAB1-GFP(+) in which both *PAB1* copies present in the genome and in the plasmid were fused with GFP. Moreover, Edc3-mCherry was used as P-bodies marker in both strains^25^. Cells grown in minimal medium till the exponential phase were exposed for 2 hours at 46 °C to induce SG and P-bodies formation, as reported by Grousl *et al.* (2009)^27^. Heat stress was selected as a representative stressful condition for SG and P-bodies analysis. Cells were analyzed under the fluorescence microscope before, during, and after heat stress. In the absence of stress, the fluorescence associated to Pab1 and Edc3 was diffused mostly in the cytoplasm in both strains, suggesting that no SG or visible P-bodies were present (Fig. 3a).

After 2 hours of incubation at 46 °C, SG and P-bodies were clearly visible in both strains (Fig. 3b). Whereas for P-bodies no relevant differences between strains were detectable, SG were brighter and apparently larger in the Pab1-GFP(+) strain (Fig. 3b). After 20 minutes of recovery at 30 °C SG and P-bodies were almost completely dissolved in the control strain, while both aggregates were still visible in Pab1-GFP(+) strain (Fig. 3c). Being SG and P-bodies dynamically linked sites through which mRNP are exchanged^24^,^25^ (as visible by the overlap between P-bodies and SG, Fig. 3b), the slower P-bodies dissolution could be related to the enduring presence of SG. It has been observed, indeed, that P-bodies disassemble slower than SG when cells are enabled to re-enter growth from stationary phase^36^.

These results show that the modulation of *PAB1* expression affects SG morphology, possibly leading to the observed increased robustness against stress, and further support the crucial role of SG in stress response. Therefore, *PAB1* emerges as a target for eliciting robust phenotypes. Our attention was focused on acetic acid, as stressor agent of lignocellulosic-based bio-processes^5^, for setting up a screening of a library of Pab1 variants.

### Selection of *PAB1* variants conferring an improved acetic acid phenotype to *S. cerevisiae*

Mutant alleles were created through random mutagenesis of *PAB1* promoter and coding sequence by error-prone PCR. The yeast screening was performed in the *S. cerevisiae* CEN.PK background containing the wild type chromosomal copy of *PAB1*. Cells were transformed either with the YCplac33*PAB1* plasmid or with the obtained mutant library and the resulting yeast library was screened in the presence of increasing concentrations of acetic acid (20, 50, 60, 70 and 80 mM) at pH 3.0 on minimal medium agar plates. The number of colonies (CFU) grown under selective pressure was compared between the yeast mutant library and the Pab1(+) strain, here used as control (Table 1).

**Figure 4:**
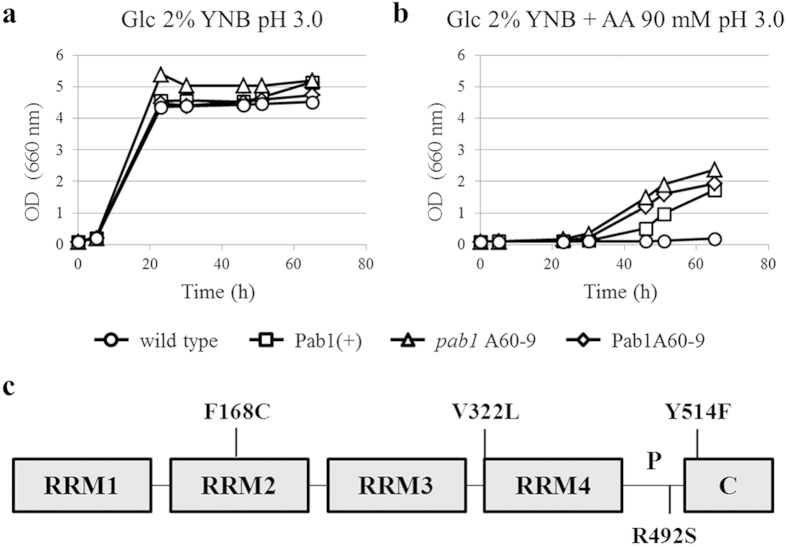
Acetic acid tolerance and Pab1 substitutions in the *pab1* A60-9 mutant strain. Wild type, Pab1(+), Pab1A60-9 strains and mutant *pab1* A60-9 were grown in minimal medium at pH 3.0 (**a**) in the absence and (**b**) in the presence of 90 mM acetic acid. The results of one representative experiment of three are shown. (**c**) Amino acid substitutions in the *pab1* A60-9 mutant are shown on a schematic representation of Pab1 protein composed of six different functional domains. AA: acetic acid; RRM: RNA recognition motif; P: proline-rich domain; C: C-terminal domain.

**Figure 5:**
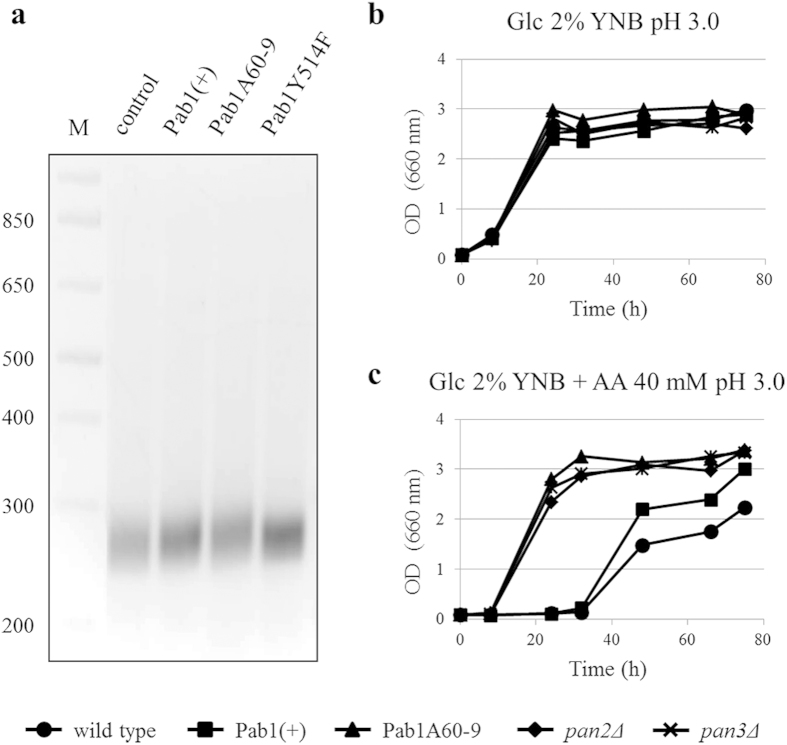
Analysis of *PGK1* mRNA poly(A) tail length and effect of the lack of PAN activity on *S. cerevisiae* tolerance to acetic acid. (**a**) Comparison of *PGK1* mRNA poly(A) tail length between wild type, Pab1(+), Pab1A60-9 and Pab1Y514F strains. PCR reactions (12.5 μl) were resolved on 3% agarose gel. Wild type, Pab1(+), Pab1A60-9, *pan2Δ* and *pan3Δ* BY4741 strains were grown in minimal medium at pH 3.0 (**b**) in the absence and (**c**) in the presence of 40 mM acetic acid. The amino acids histidine, leucine and methionine (50 mg/l) were supplemented. AA: acetic acid, M: DNA ladder. The results of one representative experiment of three are shown.

Interestingly, the number of CFU obtained by transforming cells with the *PAB1* mutant library was always higher with respect to the control. This result was not expected, given that mutations are generally detrimental or neutral, and it deserves future consideration. Since the growth of the control strain was partially and totally inhibited in the presence of 60 and 70 mM acetic acid respectively, we considered the 43 mutant clones selected in the presence of these acid concentrations as the most promising for further analyses. These mutants were analyzed in 96 multiwell plates for growth in minimal medium at pH 3.0 in the absence or in the presence of 60 and 80 mM acetic acid, together with Pab1(+) strain as control. Mutants exhibiting a better growth than the control at both acetic acid concentrations (see supplementary Fig. S2 online) were selected and successively grown in shake flasks in minimal medium at pH 3.0 in the absence or presence of 90 mM acetic acid. The mutant *pab1* A60-9 resulted as the best performing strain (see supplementary Fig. S3 online). *S. cerevisiae* CEN.PK background was then transformed with the corresponding centromeric plasmid YCplac33*A60-9* to validate that the improved phenotype was effectively conferred by the mutations identified in the mutant *pab1* A60-9. As showed in Fig. 4b, the growth of this strain (named Pab1A60-9) was similar to that of the *pab1* A60-9 mutant selected during the screening. Moreover, the improved acetic acid tolerance given by the selected mutations was confirmed in the BY4741 yeast background (Fig. 5c).

### Characterization of the mutant *pab1* A60-9

The promoter sequence of A60-9 variant contained two mutations localized in different binding sites of the putative transcription factor RC2 predicted *in silico* by Alggen PROMO software V 3.0.2^37^,^38^ (see supplementary Fig. S4 online). One silent and four missense point mutations were found in the coding sequence (see supplementary Fig. S5 online), resulting in the alteration of the amino acid sequence of Pab1 in correspondence of the residues F168, V322, R492 and Y514, located in different domains of the protein (Fig. 4c).

Interestingly, the mutation in the C-terminal region involves a residue (Y514) implicated in the interaction of Pab1 with Pan3, an essential subunit of the Pan2-Pan3 poly(A)-ribonuclease complex (PAN), which participates in the shortening of poly(A) tails^39^,^40^. Mangus *et al.* (2004) reported that the substitution of the tyrosine 514 to cysteine reduces Pab1 interaction with Pan3^40^, suggesting that mutations in this residue might negatively affect the Pab1-dependent PAN activity. In *S. cerevisiae pan2Δ* and *pan3Δ* strains mRNAs with longer poly(A) tails have been described, compared to wild type cells^40^. To verify a possible defect in the PAN-dependent mRNA deadenylation due to mutation Y514F, the poly(A) tail length of *PGK1* mRNA, suggested as reference transcript by Brown and Sachs (1998)^41^, was then compared between wild type, Pab1(+) and Pab1A60-9 strains using a PCR-based method (USB® Poly(A) Tail-Length Assay Kit, Affymetrix, see Methods). Once the cDNA was obtained from the total RNA extract, the poly(A) tail length was estimated using a forward primer specific for the 3′-UTR region and a reverse one specific for the poly(A) tail. No marked differences between the wild type and Pab1(+) strains were detected, suggesting that the sole increased expression of Pab1 does not affect the poly(A) tail length (Fig. 5a). On the contrary, in the Pab1A60-9 strain the pool of *PGK1* mRNAs appeared to have a slightly longer poly(A) tail compared with the wild type and Pab1(+) strains, as arguable from the electrophoretic shift (Fig. 5a).

To correlate longer poly(A) tails with the observed improved phenotypes, we assessed the effect of the lack of PAN activity on acetic acid resistance by analyzing the growth kinetics of *pan2Δ* and *pan3Δ* strains (BY4741 background, auxotrophic for histidine, methionine and leucine). These strains were compared to parental, Pab1(+) and Pab1A60-9 strains (reconstructed in the BY4741 background) in minimal medium at pH 3.0 in the absence or presence of acetic acid (Fig. 5b,c). In the absence of the organic acid, *pan*-deleted mutants grew similarly to the other strains (Fig. 5b). In the presence of acetic acid, however, both *pan2Δ* and *pan3Δ* strains, similarly to Pab1A60-9 strain, showed a better growth kinetic than parental and Pab1(+) strains (Fig. 5c). These results demonstrate that the lack of PAN activity increases yeast tolerance to acetic acid, which has never been shown in literature, suggesting a possible correlation between longer poly(A) tails and stress resistance.

**Figure 6:**
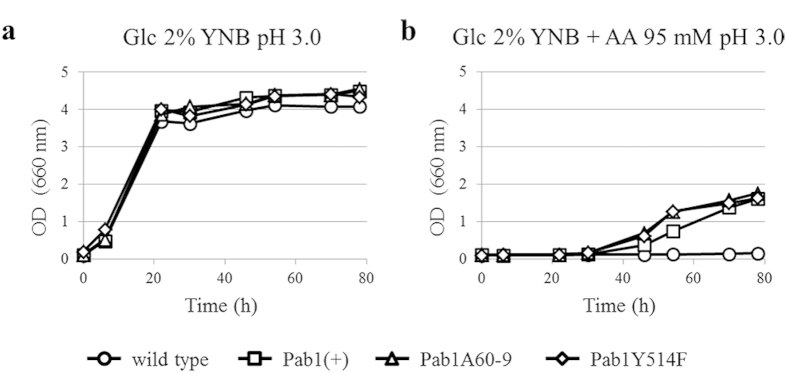
Effect of Y514F mutation on *S. cerevisiae* growth in the presence of acetic acid. Wild type, Pab1(+), Pab1A60-9 and Pab1Y514F strains were grown in minimal medium at pH 3.0 (a) in the absence and (b) in the presence of 95 mM acetic acid. AA: acetic acid. The results of one representative experiment of three are shown.

Finally, to demonstrate the direct involvement of Y514F mutation in determining longer poly(A) tails, poly(A) tail length of *PGK1* mRNA was analyzed in the Pab1Y514F strain, which contains the genomic wild type copy of *PAB1* and the plasmid YCplac33*PAB1*^*Y514F*^. According to our speculations, also this strain exhibited a pool of mRNAs with longer poly(A) tails (Fig. 5a). Indeed, the growth kinetics of Pab1Y514F and Pab1A60-9 strains in the presence of acetic acid were completely overlapped (Fig. 6b). These results evidence a strong correlation between the mutation localized at the C-terminal, and the resulting longer poly(A) tails, and the stress tolerant phenotype showed by Pab1A60-9 strain.

### Y514F affects SG assembly during heat stress

**Figure 7:**
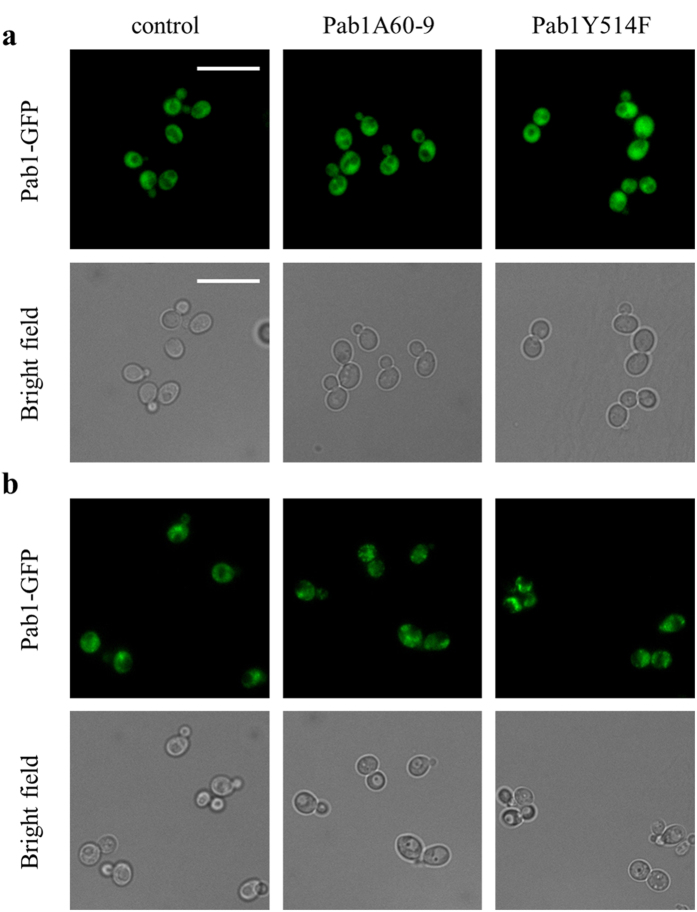
SG morphology in Pab1 mutant *S. cerevisiae* strains. Pab1 associated fluorescence of wild type (control), Pab1A60-9 and Pab1Y514F strains were analyzed (**a**) in the absence of stress and (**b**) after incubation at 46 °C for 2 h. Scale bar, 10 μm.

The analysis of SG morphology before and after 2 hours of exposure at 46 °C was repeated in the strains Pab1A60-9 and Pab1Y514F, carrying the genomic version of Pab1 tagged with GFP and the plasmid YCplac33*A609* or YCplac33*PAB1*^*Y514F*^, respectively (Fig. 7). In this setting, only the tagged native protein was used as a marker to allow the comparison of SG morphology between mutants and the control strain.

In the absence of the stress, fluorescence associated to Pab1 was diffused within cells in all strains (Fig. 7a), in agreement with the experiment reported in Fig. 3. After 2 hours of exposure at 46 °C, SG were smaller and more scattered in both mutant strains compared to the control one (Fig. 7b). This correlation indicates that the variation in SG assembly observed in the Pab1A60-9 strain is likely due to the mutation Y514F.

## Discussion

Understanding the key mechanisms involved in stress response allows the identification of elements whose alteration can ameliorate cell factories performances. In this scenario, the tuning of the existing mRNAs pool represents one of the promptest cellular responses to face stressful conditions^42^. The modulation of factors involved in the control of mRNA fate has therefore the potential for the development of robust cell factories. Indeed, here we demonstrate that the modulation of Pab1 expression improves *S. cerevisiae* stress tolerance. Although further researches will help to elucidate the reasons of the ameliorated phenotype, we speculate that it could be at least partially related to the altered SG morphology, and especially with the longer persistence of these structures, which offer a storage compartmentalization for mRNA. Moreover, the screening of a mutant *pab1* library allowed the selection of *pab1* A60-9 showing a further improved tolerance to acetic acid. The version of Pab1 responsible of the ameliorated phenotype harbors four amino acid substitutions, F168C, V322L, R492S, Y514F, located in different functional domains of the protein (see again Fig. 4c). F168 is a RNA-binding residue and mutations in this site, in particular into cysteine, were indicated as deleterious for Pab1 function by a deep mutational scanning of the RRM2 domain^43^. However, our results seem in contradiction with that. This discordance can be ascribed to the different approaches and genetic backgrounds used to characterize or select this mutation. The deep mutational scanning of RRM2 was performed in strains lacking the chromosomal wild type *PAB1* gene, whereas in the mutant *pab1* A60-9 the endogenous version of Pab1 could compensate the deleterious effect of F168C. Moreover and contrary to the present study, in Melamed *et al.* (2013)^43^ the association of F168C with growth impairment was determined by using a truncated version of Pab1, harboring the RRM1-RRM2-RRM3 domains and only 25 amino acids of the N-terminal RRM4 domain. Therefore, we cannot exclude that the effect of F168C mutation on full-length Pab1 functionality might be different. This hypothesis is supported by the evidence that the mutation F170V, which injured cell growth when present in the truncated form of the protein, but not in the full-length one, was also identified and described in the same study^43^. Finally, we cannot exclude that the other mutations could compensate for the possible defect of the first one. V322L is localized at the beginning of the RRM4 domain, which has been described to interact with proteins implied in mRNA export from the nucleus, and R492S is in correspondence of the P domain, which is critical for Pab1 self-association and mediates poly(A) tail deadenylation performed by the Ccr4-NOT complex^44^,^45^. However, the specific role of these two residues has not been yet characterized. Y514, localized at the C-terminal domain of the protein, has been reported to be involved in Pab1 interaction with the Pan3 subunit of PAN complex, promoting the deadenylation activity^39^,^40^. *pan2Δ* or *pan3Δ* mutants, both defective in PAN function, display mRNAs with longer poly(A) tails compared to wild type strains^39^,^40^. The longer *PGK1* poly(A) tails found in Pab1A609 and Pab1Y514F strains suggest therefore that the amino acid substitution in the C-terminal domain of the mutant Pab1 version impairs the interaction with Pan3, decreasing the rate of global deadenylation. It is important to underline that the poly(A) tail length has a prominent role in transcript stability and translational stimulation, and, in general, its shortening turns out in mRNA decay and decreased translation^46^,^47^. Concurring, a correlation between defective deadenylation and mRNA stabilization has been described^48^. The presence of a pool of mRNAs with a longer poly(A) tail, and so stabilized, might therefore promote a faster cellular recovery from the stress-induced damage compared to wild type cells, leading to the growth advantage shown by Pab1A609 and Pab1Y514F strains in the presence of acetic acid (Fig. 6b). Thus, the mutation Y514F significantly contributes to such phenotype. Furthermore, we demonstrate that this mutation also causes alterations in SG morphology, suggesting that it might determine changes in mRNA metabolism regarding both the control of poly(A) tail length and SG formation. Although the correlation between mutations and SG morphology needs to be further elucidated, to our knowledge this is the first report of a putative implication of the residue F514 of Pab1 in SG morphology in *S. cerevisiae*.

Overall, we demonstrated that *PAB1* represents a powerful target for the rewiring of *S. cerevisiae* into stress tolerant strains. We believe that Pab1 modulation alters global gene expression at post-transcriptional level, as suggested by SG and poly(A) tail length analysis, leading to complex phenotypes difficult to obtain through rational design. For these reasons, the here proposed method can represent an innovative approach for the development of robust cell factories, which are required for the implementation of industrial bioprocesses. In this perspective, the performance of the obtained yeast strains, in terms of multiple stresses tolerance and production, is currently under evaluation in conditions similar to those occurring during industrial fermentations. In fact, differently from the conditions used to select the mutant *pab1* A60-9, industrial processes take generally place at pH near 5.0 and in the presence of various inhibitors^5^. In this work, pH 3.0 was selected to promote the presence of the more toxic undissociated form of the acid in the screening medium, and so to have a strong selective pressure. Moreover, the role of Pab1 C-terminal region in stress response and resistance is under investigation, considering the possibility to utilize designed parts of Pab1 protein for tuning *S. cerevisiae* robustness.

It is important to underline that the described modifications might not have the same effects in industrial yeasts, which are generally more robust than laboratory strains, here used. In particular, industrial strains could have an inherently greater expression level of *PAB1*, possibly limiting the application of the described strategy to improve their robustness. Nevertheless, in this work we obtained successful results using the BY4741 strain, in which Pab1 has been demonstrated to be one of the 50 most expressed proteins^49^, suggesting that Pab1 modifications are effective also in the presence of high *PAB1* expression levels. For these reasons, we are currently evaluating whether the ameliorated phenotypes induced by Pab1 modifications can be transferred to the robust industrial Ethanol Red strain, which will be further characterized for its metabolic traits. Indeed, the creation of a robust cell factory is reliable only when the introduced modifications do not impair indispensable traits for its industrial use. As an example, the xylose-fermenting strain GS1.11-26 was obtained by metabolic and evolutionary engineering of the industrial strain Ethanol Red, but had a partial respiratory defect and reduced ethanol accumulation in very high-density fermentations compared to the parental strain^50^. Subsequently, three superior strains were obtained from GS1.11–26 through its mating or meiotic recombination with other strains followed by the selection of the desired phenotypes^51^.

Furthermore, the correlation found between Pab1 mutations and abnormal SG morphology makes the here described yeast mutants potential models for the study of human SG-associated neurodegenerative diseases, such as Alzheimer and Amyotrophic Lateral Sclerosis (ALS)^52^. Pab1 mutations affecting SG assembly may allow the identification of novel biological associations and promising therapeutic targets.

In conclusion, at the best of our knowledge, the presented work shows for the first time the development of robust strains through the modulation of a SG component in *S. cerevisiae*. We retain that, in addition to Pab1, other key proteins involved in SG assembly, or more generally in mRNA cycle, represent potential candidates for the development of strains with improved features.

## Methods

### Yeast strains, media, growth conditions

The *S. cerevisiae* genetic backgrounds used in this study were BY4741 (*MATa*; *his3Δ0; leu2Δ0; met15Δ0; ura3Δ0*) (EUROSCARF), CEN.PK113-5D (*MATa*; *MAL2-8c*; *SUC2*; *ura3-52*) and CEN.PK113-11C (*MATa*; *MAL2-8c*; *SUC2*; *ura3-52*; *his3Δ1*)^53^,^54^. Yeast transformations were performed according to the LiAc/PEG/ss-DNA protocol^55^. The *S. cerevisiae* strains constructed in this study are listed in Supplementary Table S1, with their respective genotypes.

The oligonucleotide pairs used are listed in Supplementary Table S2. To delete the *EDC3* gene, the *edc3*::hphMX4 cassette was amplified from the plasmid pAG26^56^, using the oligonucleotides ΔEdc3 fw and ΔEdc3 rev. The *edc3*::hphMX4 cassette was used to replace *EDC3* gene in CEN.PK113-11C strain. Positive clones were selected on YPD agar plates supplemented with the antibiotic hygromycin B (Roche) to the final concentration of 2 mg/ml. Gene disruption was confirmed by PCR analysis using the oligonucleotides pairs Edc3 fw/Hph rev and Hph fw/Edc3 rev.

The chromosomal GFP-tagged version of Pab1 was created using the cassette *PAB1-GFP*::His3MX6 amplified from the plasmid pFA6a-GFP(S65T)-His3MX6^57^, using the oligonucleotides Pab1 GFP fw and Pab1 GFP rev. The PCR-amplified fragment was directly used for yeast transformation.

Yeast cultures were grown in minimal synthetic medium (0.67% YNB Biolife without amino acids) with 2% w/v D-glucose as carbon source. When required, supplements such as histidine, leucine, methionine and uracil (Sigma) were added to the final concentration of 50 mg/l. Growth kinetics in the presence of acetic acid were performed at pH 3.0 to promote the presence of the more toxic undissociated form of the acid in the broth culture. Different concentrations of acetic acid were used for the two yeast genetic backgrounds (40 mM for BY4741, 90 mM for CEN.PK) because of their different intrinsic resistance^58^.

For the determination of growth kinetics, yeast cells were inoculated at an initial optical density of 0.1 (OD_660_) and then the OD was measured at specific time intervals over at least 78 hours from the inocula. For each experiment, independent biological triplicates have been executed. In figures, the results of one representative experiment are shown. All strains were grown in shake flasks at 30 °C, unless otherwise stated, and 160 rpm. The ratio of flask volume:medium was 5:1.

### Gene amplification and expression plasmid construction

The *PAB1* endogenous ORF and the native promoter region were PCR amplified from genomic DNA, isolated from S288c *S. cerevisiae* strain, on a GeneAmp PCR System 9700 (PE Appl. Biosystems, Inc.) using Q5^®^ High-Fidelity DNA Polymerase (New England Biolabs) and following the manufacturer’s instructions. The amplification was performed using the oligonucleotides Pab1 PstI fw and Pab1 SalI rev. The PCR product was *Pst*I and *Sal*I cut and sub-cloned in the low copy number expression vector pYF8 digested with the same restriction enzymes to excide the TPI promoter. To create the plasmid pYF8, the DNA sequence corresponding to the TPI promoter-multi cloning site-poly(A) terminator was amplified using the oligonucleotides TPIp PstI fw and TPIp EcoRI rev from the integrative plasmid pYX042(-ATG), previously obtained from the pYX042 integrative expression vector (R&D Systems) (*Eco*RI and *Bam*HI cut, blunt-ended and re-legated). The PCR-product was cut with *Pst*I and *Eco*RI and sub-cloned in the plasmid YCplac33 (centromeric, *URA3* auxotrophic marker)^59^.

The oligonucleotides Pab1 GFP PstI fw and Pab1 GFP SalI rev were used to amplify the chromosomally integrated GFP-tagged version of Pab1. The PCR product was *Pst*I and *Sal*I cut and sub-cloned in the pYF8 plasmid to obtain the plasmid YCplac33*PAB1-GFP*.

The mutated *PAB1* version was *Pst*I and *Sal*I cut from the plasmid isolated from the mutant strain *pab1A60-9* and sub-cloned into the pYF8 to create the plasmid YCplac33*A60-9*.

The plasmid YCplac33*PAB1*^*Y514F*^*-GFP* was obtained using the Gibson Assembly Cloning Kit (New England Biolabs, catalog #E5510S), following the manufacturer’s instruction. Two fragments with overlapping ends were amplified from the plasmid YCplac33*PAB1-GFP* using the oligonucleotides Pab1 Y514F fw and Pab1 GFP SalI rev, or Pab1 PstI fw and Pab1 Y514F rev. The two PCR-derived inserts were then assembled with the YCplac33 backbone cut from the plasmid YCplac33*PAB1-GFP* with *Pst*I and *Bss*HII.

The *PAB1*^*Y514F*^ sequence was amplified from the plasmid YCplac33*PAB1*^*Y514F*^*-GFP* using the oligonucleotides Pab1 PstI fw and Pab1 SalI rev and sub-cloned in the pYF8 plasmid to obtain the plasmid YCplac33*PAB1*^*Y514F*^.

All the restriction enzymes used were from New England Biolabs (Hitchin, Herts, UK). Standard procedures were employed for all cloning purposes^60^.

The plasmids pRP1574 (Edc3-mCh, centromeric, *URA3* auxotrophic marker) and pRP1657 (Pab1-GFP, Edc3-mCh; centromeric, *URA3* auxotrophic marker) were kindly provided by Prof. Parker^25^.

### Total protein extraction and western blot analysis

10^8^ exponentially growing cells were disrupted with glass beads (425–600 μm, Sigma) in 20% TCA. After centrifugation, pellets were resuspended in Leammli sample buffer (2% SDS, 10% glycerol, 5% β-mercaptoethanol, 0.02% bromophenol blue, Tris-HCl 60 mM pH 6, 8) and in TRIS 1 M (pH 7.0). Samples were boiled for 3 min, centrifuged and the supernatants collected for western blot analysis. Equal volumes of total protein extracts (15 μl) were resolved in a SDS-polyacrylamide gel electrophoresis and transferred on a nitrocellulose membrane (blocked with 5% milk in TBS-Tween overnight at 4 °C). Pab1-GFP was detected using a monoclonal anti-GFP antibody (Living Colors A.v JL-8, Diatech labline, 1:1000 in TBS-Tween-5% milk for 2 hours). Actin was detected using a monoclonal anti-actin antibody (Abcam 2Q1055, 1:1000 in TBS-Tween-5% milk for 3 hours). The membranes were washed in TBS-Tween and incubated with Rabbit anti-Mouse IgG (FC) secondary antibody, AP (alkaline phosphatase) conjugate diluted 1:15000 in TBS-Tween-5% milk for 1 hour at RT. The membranes were developed adding CDP-Star Chemiluminescent Substrate (Sigma) for 5 min at RT under gentle agitation and then exposed to Pierce CL-Xposure film to reveal Pab1-GFP and actin signals. Bands were quantified using ImageJ 1.47v software.

### Mutant library construction

The library was created using the GeneMorph II EZClone Domain Mutagenesis Kit (Agilent Technologies, catalog #200552) following the manufacturer’s instructions. Briefly, mutations in the *PAB1* promoter and coding sequence were introduced through error-prone PCR using the Mutazyme II DNA polymerase provided by the kit. The oligonucleotides used for this amplification were Pab1 mut fw and Pab1 mut rev. 250 ng of the plasmid YCplac33*PAB1* were used as DNA template in order to produce a medium mutational frequency (7.43 mutations/kb) according to manufacturer’s instructions. Following purification, the resulting mutated PCR products were used as “megaprimers” for the EZClone reaction, during which they were denaturated and annealed to the original donor plasmid and extended with a specialized enzyme mix containing a high fidelity DNA polymerase. The plasmid library was transformed into *Escherichia coli* XL10-Gold ultracompetent cells that were plated onto LB-ampicillin agar plates (antibiotic concentration of 100 mg/l). The library size was approximately 6 × 10^3^. Plasmid DNA was isolated from *E. coli* using the alkaline lysis method^60^ and transformed into CEN.PK yeast background. Yeast cells were scraped off the plates, inoculated in minimal medium and incubated for about 6 hours at 30 °C and 160 rpm. This liquid culture was used to create frozen glycerol stocks (80% yeast culture, 20% glycerol) that were conserved at -80 °C.

### Phenotype selection

The yeast glycerol stocks were inoculated in minimal medium and incubated for about 4-6 hours at 30 °C at 160 rpm. Successively, to cover the library at least three times, cell cultures were diluted to the final OD_660_ of 0.001 and 750 μl were plated onto minimal medium agar plates, containing increasing concentrations of acetic acid (20, 50, 60, 70 and 80 mM) at pH 3.0. Cells were also plated on minimal medium agar plates in the absence of stress in order to verify that an equal amount of cells transformed with the control plasmid and the mutant library were plated, and also to check whether the whole library was plated. Plates were incubated at 30 °C for 3 days and the colony forming units (CFU) were counted. The growth kinetic of the isolated clones was tested in the presence of acetic acid 60 and 80 mM at pH 3.0 in 96 multiwell plates. Cells were inoculated, in duplicate, at an initial OD_660_ between 0.018 and 0.025 in each well. Multiwell plates were then incubated at 30 °C and 1250 rpm and then the OD_660_ was measured at specific time intervals over at least 70 hours from the inocula.

### Poly(A) tail-length assay

Total RNA was extracted from exponentially growing cells using the Aurum^TM^ Total RNA Mini Kit (Biorad, catalog #732-6820). Poly(A) tail-length was determined using the USB® Poly(A) Tail-Length Assay Kit (Affimetrix, catalog #76455) following the manufacturer’s instructions. The gene-specific forward oligonucleotide Pgk1 fw was used for the poly(A) tail-length analysis of *PGK1* mRNAs. PCR products were resolved on 3% agarose gel using Novel Juice for the visualization of DNA bands upon UV illumination.

### Fluorescence microscopy analysis

Stress granules and P bodies were analyzed in cells grown in minimal medium till the exponential phase (OD_660_ = 0.4–0.6) and exposed at 46 °C for 2 hours. Cell cultures were then shifted at 30 °C for 20 minutes to recover from heat stress. During the whole experiment, about 10^8^ cells were collected, resuspended in PBS (NaH_2_PO_4_ 53 mM, Na_2_HPO_4_ 613 mM, NaCl 75 mM) and then observed in a Nikon ECLIPSE 90i fluorescence microscope (Nikon) equipped with a 100X objective. Emission fluorescence due to Pab1-GFP or Edc3-mCh was detected by B-2A (EX 450-490 DM 505 BA520) or G-2A (DX 510-560 DM 575 BA 590) filter (Nikon), respectively. Digital images were acquired with a CoolSnap CCD camera (Photometrics) using MetaMorph 6.3 software (Molecular Devices). The images were merged using Adobe Photoshop CC.

## Additional Information

**How to cite this article**: Martani, F. *et al.* The *Saccharomyces cerevisiae* poly(A) binding protein Pab1 as a target for eliciting stress tolerant phenotypes. *Sci. Rep.* **5**, 18318; doi: 10.1038/srep18318 (2015).

## Supplementary Material

Supplementary Information

## Figures and Tables

**Table 1:** Numbers of colony forming units (CFU) obtained from the screening in the presence of acetic acid.

	**Acetic acid pH 3.0**				
	20 mM	50 mM	60 mM	70 mM	80 mM
Control	1621 CFU	592 CFU	18 CFU	0 CFU	0 CFU
Mutant library	2412 CFU	1165 CFU	32 CFU	11 CFU	0 CFU

CEN.PK cells transformed with the control plasmid (YCplac33*PAB1*) or the *PAB1* mutant library were plated onto minimal medium agar plates containing increasing concentrations of acetic acid (20, 50, 60, 70 and 80 mM) at pH 3.0. CFUs were counted after 3 days of growth at 30 °C.
