# Supplementary material for: The Saccharomyces cerevisiae poly(A) binding protein Pab1 as a target for eliciting stress tolerant phenotypes
Source: Sci Rep. 2015 Dec 14;5:18318. doi: 10.1038/srep18318 (PMC4677312; doi:10.1038/srep18318)
Supplement: Supplementary Information [file srep18318-s1.pdf]

The *Saccharomyces cerevisiae* poly(A) binding protein Pab1 as a target for eliciting stress tolerant phenotypes

Francesca Martani, Francesca Marano, Stefano Bertacchi, Danilo Porro and Paola Branduardi

Department of Biotechnology and Biosciences, University of Milano Bicocca, Piazza della Scienza 2, 20126 Milano (Italy)

SUPPLEMENTARY INFORMATION

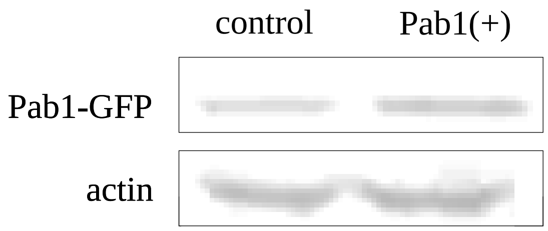

Supplementary Figure S1. Western blot analysis of Pab1-GFP and actin levels in control and Pab1(+)-GFP strains. Anti-GFP and anti-actin were used as primary antibodies, and AP conjugated rabbit anti-Mouse IgG (FC) as the secondary antibody.

A

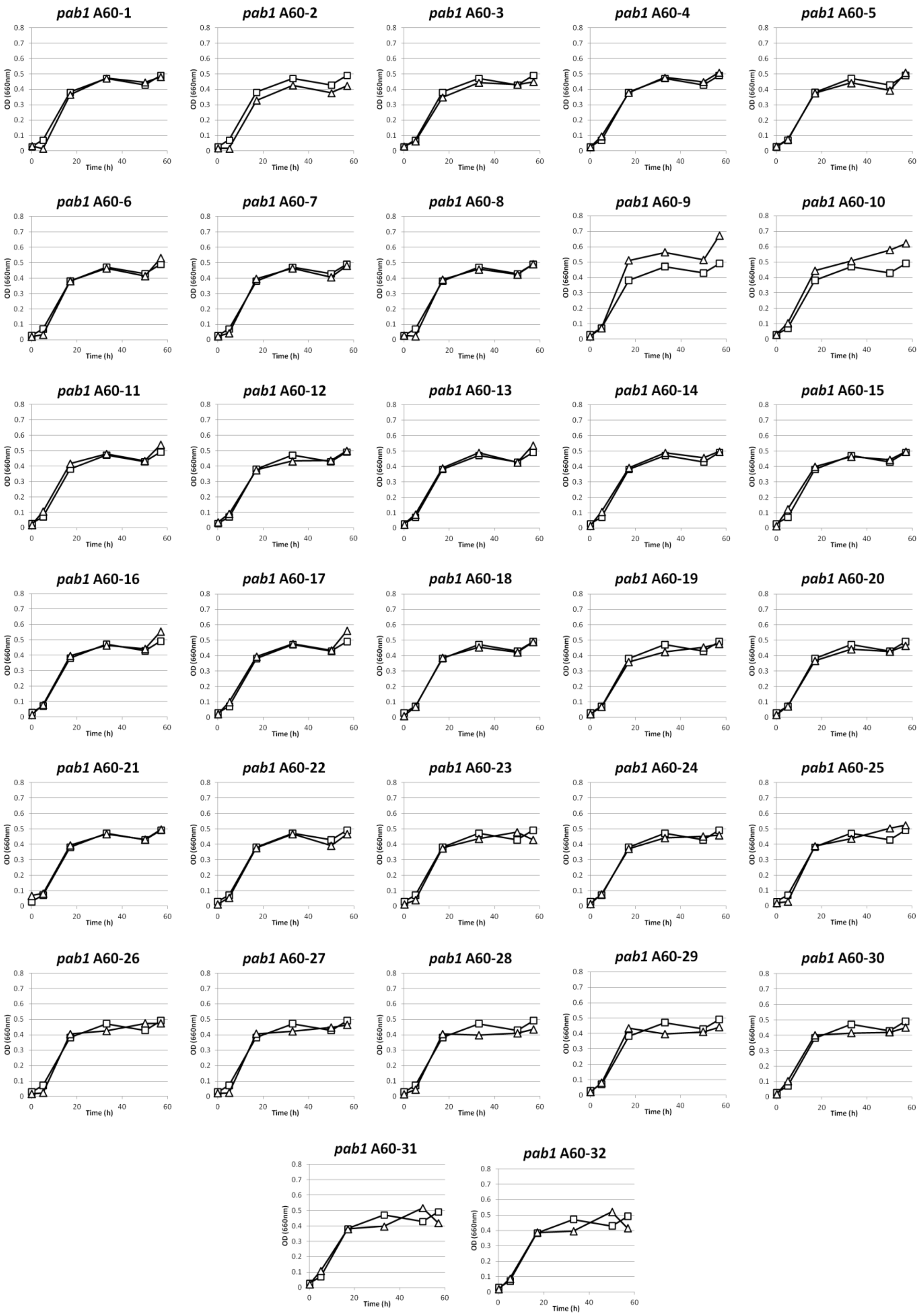
pab1 A60-1
pab1 A60-2
pab1 A60-3
pab1 A60-4
pab1 A60-5
pab1 A60-6
pab1 A60-7
pab1 A60-8
pab1 A60-9
pab1 A60-10
pab1 A60-11
pab1 A60-12
pab1 A60-13
pab1 A60-14
pab1 A60-15
pab1 A60-16
pab1 A60-17
pab1 A60-18
pab1 A60-19
pab1 A60-20
pab1 A60-21
pab1 A60-22
pab1 A60-23
pab1 A60-24
pab1 A60-25
pab1 A60-26
pab1 A60-27
pab1 A60-28
pab1 A60-29
pab1 A60-30
pab1 A60-31
pab1 A60-32
OD (660nm)
Time (h)

B

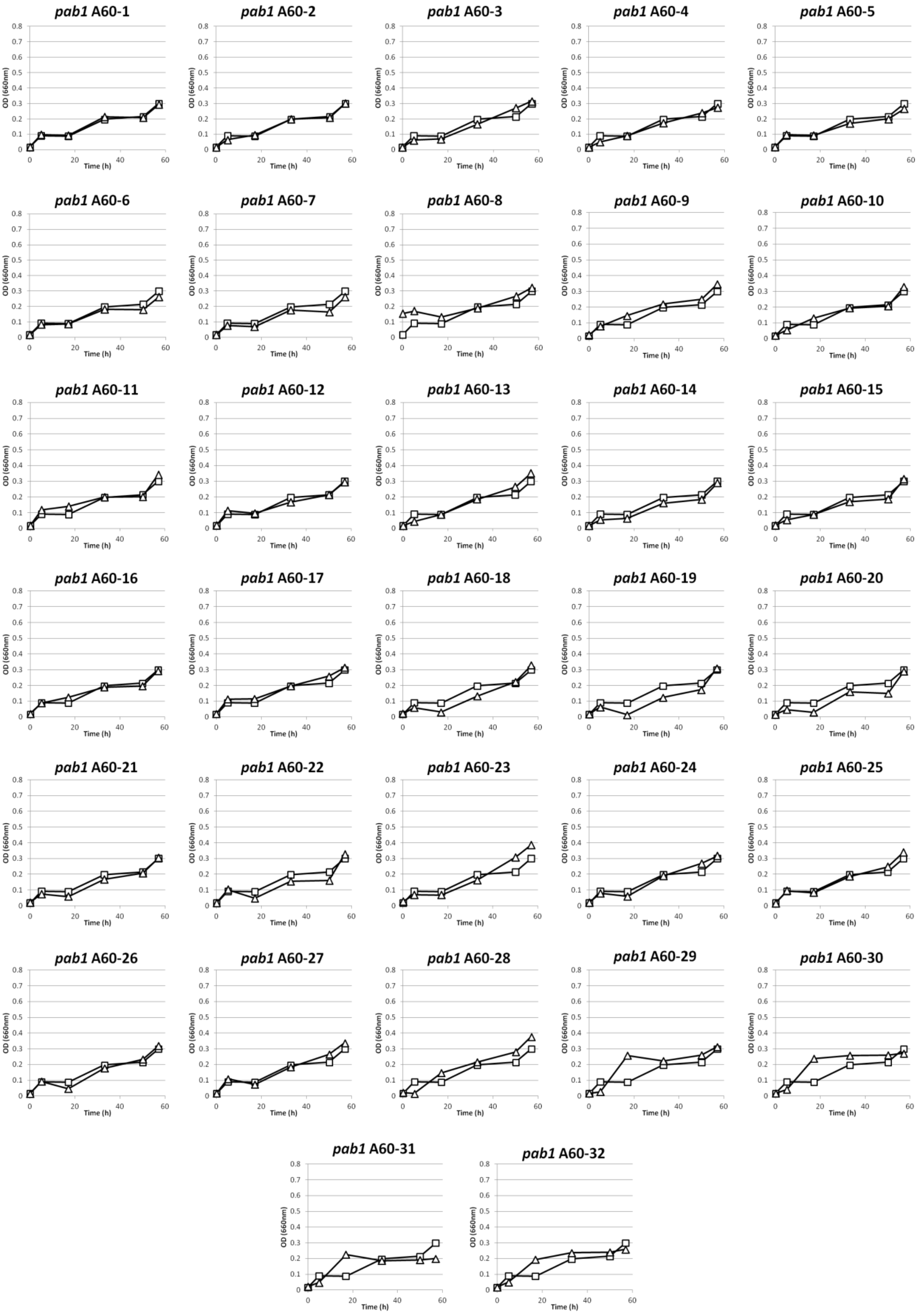
pab1 A60-1
pab1 A60-2
pab1 A60-3
pab1 A60-4
pab1 A60-5
pab1 A60-6
pab1 A60-7
pab1 A60-8
pab1 A60-9
pab1 A60-10
pab1 A60-11
pab1 A60-12
pab1 A60-13
pab1 A60-14
pab1 A60-15
pab1 A60-16
pab1 A60-17
pab1 A60-18
pab1 A60-19
pab1 A60-20
pab1 A60-21
pab1 A60-22
pab1 A60-23
pab1 A60-24
pab1 A60-25
pab1 A60-26
pab1 A60-27
pab1 A60-28
pab1 A60-29
pab1 A60-30
pab1 A60-31
pab1 A60-32
OD (660nm)
Time (h)

C

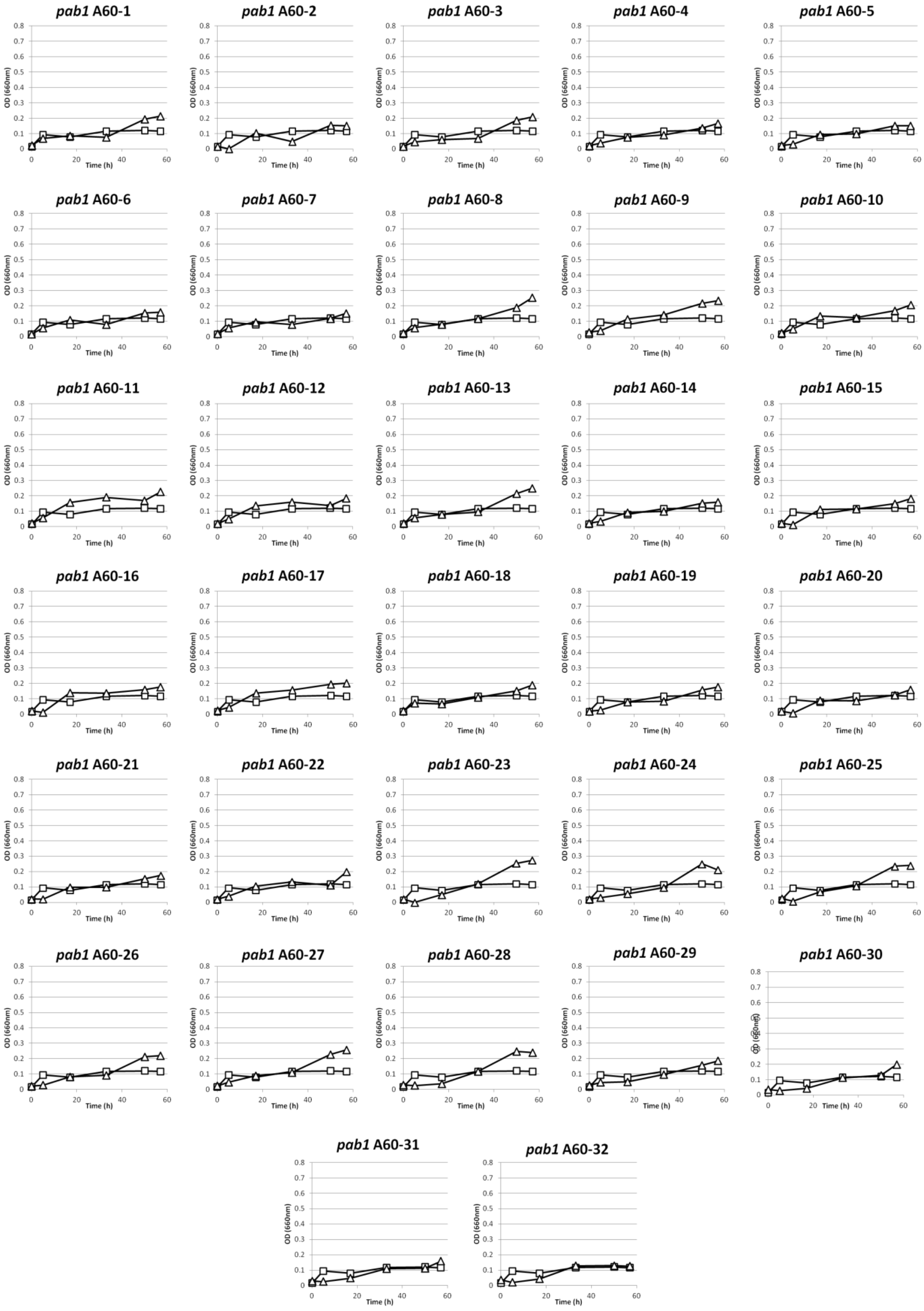

pab1 A60-1
pab1 A60-2
pab1 A60-3
pab1 A60-4
pab1 A60-5
pab1 A60-6
pab1 A60-7
pab1 A60-8
pab1 A60-9
pab1 A60-10
pab1 A60-11
pab1 A60-12
pab1 A60-13
pab1 A60-14
pab1 A60-15
pab1 A60-16
pab1 A60-17
pab1 A60-18
pab1 A60-19
pab1 A60-20
pab1 A60-21
pab1 A60-22
pab1 A60-23
pab1 A60-24
pab1 A60-25
pab1 A60-26
pab1 A60-27
pab1 A60-28
pab1 A60-29
pab1 A60-30
pab1 A60-31
pab1 A60-32
OD (660nm)
Time (h)

D

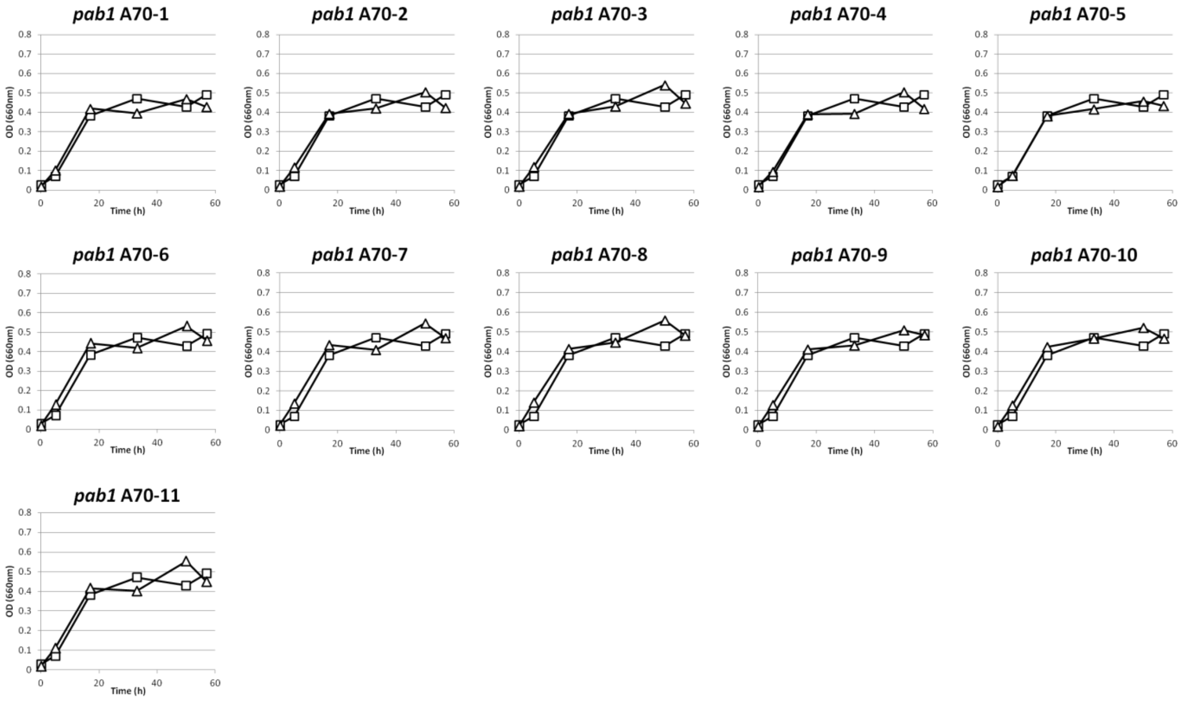
pab1 A70-1
pab1 A70-2
pab1 A70-3
pab1 A70-4
pab1 A70-5
pab1 A70-6
pab1 A70-7
pab1 A70-8
pab1 A70-9
pab1 A70-10
pab1 A70-11
OD (660nm)
Time (h)

E

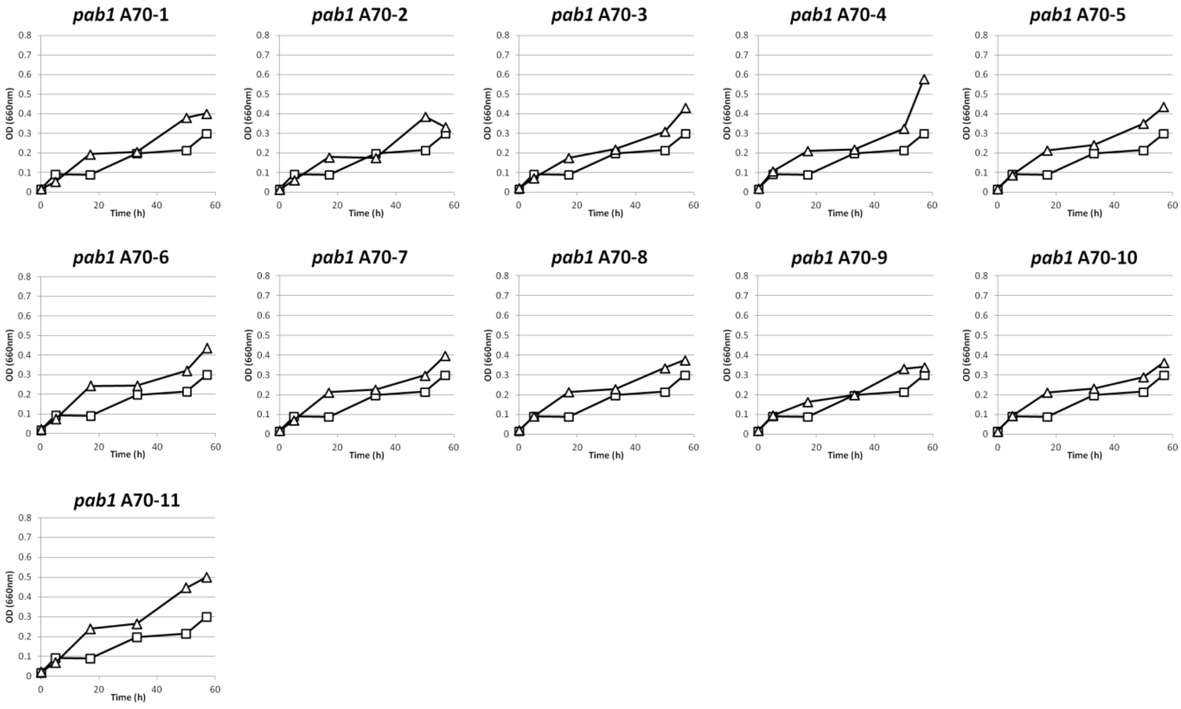
pab1 A70-1
pab1 A70-2
pab1 A70-3
pab1 A70-4
pab1 A70-5
pab1 A70-6
pab1 A70-7
pab1 A70-8
pab1 A70-9
pab1 A70-10
pab1 A70-11
OD (660nm)
Time (h)

F

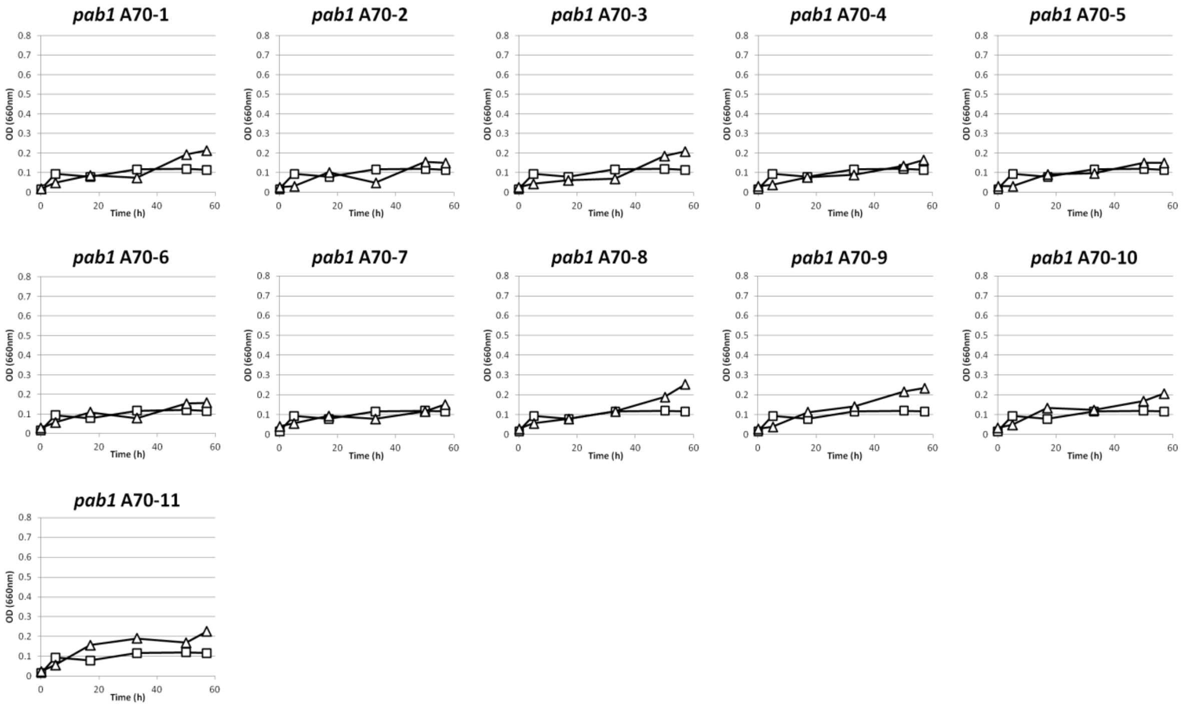

Supplementary Figure S2. Growth kinetics in multiwell plates of mutants selected on acetic acid-containing agar plates. Pab1(+) strain (square) and mutant clones (triangle) were grown in minimal medium at pH 3 in the absence (A and D) or presence of 60 mM (B and E) and 80 mM (C and F) acetic acid. The numbers reported on the top of the graphics correspond to the codex use to identify the mutants selected during the screening. 60: clones selected in the presence of 60 mM acetic acid pH 3; 70: clones selected in the presence of 70 mM acetic acid pH 3; the last is the sequential number of clones selected under the same condition. The results of one representative experiment of three are shown.

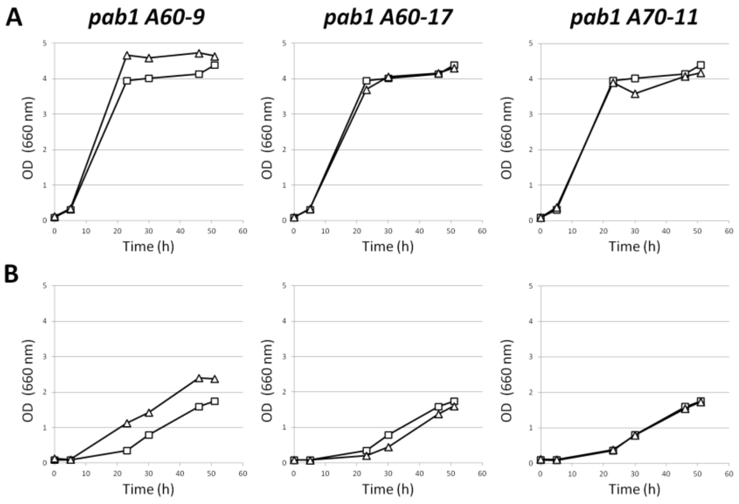

Supplementary Figure S3. Growth kinetics in shake flasks of mutants *pab1 A60-9,* pab1 *A60-17* and *pab1 A70-11.* Pab1(+) strain (square) and mutant clones (triangle) were grown in minimal medium at pH 3 in the absence (A) or presence of 90 mM (B) acetic acid. The numbers reported on the top of the graphics correspond to the codex use to identify the mutants selected during the screening. 60: clones selected in the presence of 60 mM acetic acid pH 3; 70: clones selected in the presence of 70 mM acetic acid pH 3; the last is the sequential number of clones selected under the same condition. The results of one representative experiment of three are shown.

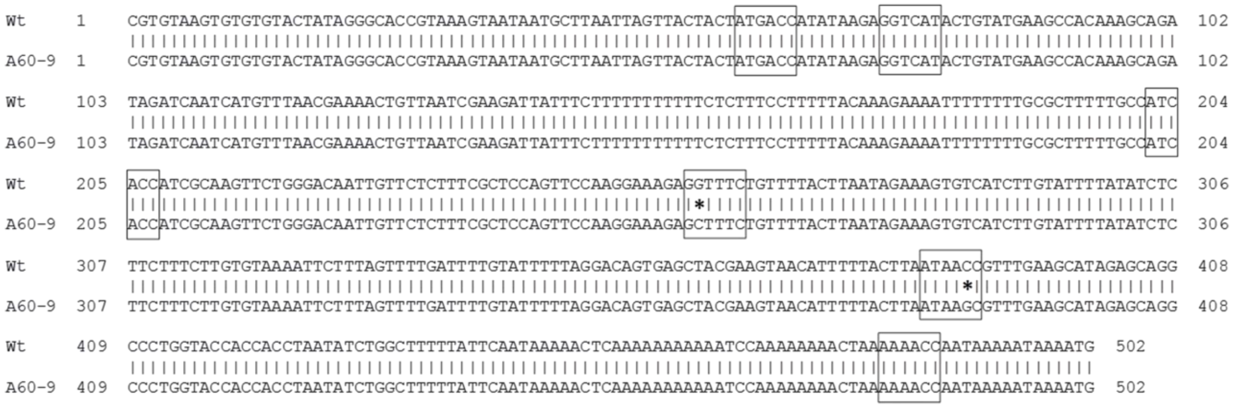

Supplementary Figure S4. Sequence alignment of the promoter region of *pab1 A60-9* mutant. The mutated nucleotides are indicated with stars. Rectangular forms indicate the binding sites of the putative transcription factor RC2.

```
PAB1 WT          1 ATGGCTGATATTACTGATAAGACAGCTGAACAATTGGAAAACTTGAATAT     50
                   ||||||||||||||||||||||||||||||||||||||||||||||||||
PAB1 A60-9       1 ATGGCTGATATTACTGATAAGACAGCTGAACAATTGGAAAACTTGAATAT     50

PAB1 WT         51 TCAAGATGACCAAAAGCAAGCCGCCACTGGTTCAGAAAGCCAATCTGTTG    100
                   ||||||||||||||||||||||||||||||||||||||||||||||||||
PAB1 A60-9      51 TCAAGATGACCAAAAGCAAGCCGCCACTGGTTCAGAAAGCCAATCTGTTG    100

PAB1 WT        101 AAAACTCTTCTGCATCATTATATGTTGGTGACTTAGAACCTTCTGTTTCC    150
                   ||||||||||||||||||||||||||||||||||||||||||||||||||
PAB1 A60-9     101 AAAACTCTTCTGCATCATTATATGTTGGTGACTTAGAACCTTCTGTTTCC    150

PAB1 WT        151 GAAGCCCACTTATATGATATCTTCTCTCCAATCGGTTCAGTCTCCTCCAT    200
                   ||||||||||||||||||||||||||||||||||||||||||||||||||
PAB1 A60-9     151 GAAGCCCACTTATATGATATCTTCTCTCCAATCGGTTCAGTCTCCTCCAT    200

PAB1 WT        201 TCGTGTCTGTCGTGATGCCATCACTAAGACCTCTTTGGGCTATGCTTATG    250
                   ||||||||||||||||||||||||||||||||||||||||||||||||||
PAB1 A60-9     201 TCGTGTCTGTCGTGATGCCATCACTAAGACCTCTTTGGGCTATGCTTATG    250

PAB1 WT        251 TTAACTTTAACGACCATGAAGCCGGCAGAAAAGCAATTGAGCAATTGAAC    300
                   ||||||||||||||||||||||||||||||||||||||||||||||||||
PAB1 A60-9     251 TTAACTTTAACGACCATGAAGCCGGCAGAAAAGCAATTGAGCAATTGAAC    300

PAB1 WT        301 TACACTCCAATCAAGGGTAGATTATGCCGTATTATGTGGTCTCAACGTGA    350
                   ||||||||||||||||||||||||||||||||||||||||||||||||||
PAB1 A60-9     301 TACACTCCAATCAAGGGTAGATTATGCCGTATTATGTGGTCTCAACGTGA    350

PAB1 WT        351 CCCATCATTGAGAAAGAAGGGTTCTGGTAACATCTTTATCAAGAACTTGC    400
                   ||||||||||||||||||||||||||||||||||||||||||||||||||
PAB1 A60-9     351 CCCATCATTGAGAAAGAAGGGTTCTGGTAACATCTTTATCAAGAACTTGC    400

PAB1 WT        401 ACCCTGATATTGACAACAAGGCTTTGTATGACACTTTCTCTGTGTTTGGT    450
                   ||||||||||||||||||||||||||||||||||||||||||||||||||
PAB1 A60-9     401 ACCCTGATATTGACAACAAGGCTTTGTATGACACTTTCTCTGTGTTTGGT    450

PAB1 WT        451 GACATCTTGTCCAGCAAGATTGCCACCGACGAAAACGGAAAATCCAAGGG    500
                   ||||||||||||||||||||||||||||||||||||||||||||||||||
PAB1 A60-9     451 GACATCTTGTCCAGCAAGATTGCCACCGACGAAAACGGAAAATCCAAGGG    500

PAB1 WT        501 TTTTGGGTTTGTTCACTTCGAAGAAGAAGGTGCTGCCAAGGAAGCTATTG    550
                   ||*|||||||||||||||||||||||||||||||||||||||||||||||
PAB1 A60-9     501 TTGTGGGTTTGTTCACTTCGAAGAAGAAGGTGCTGCCAAGGAAGCTATTG    550

PAB1 WT        551 ATGCTTTGAATGGTATGCTGTTGAACGGTCAAGAAATTTATGTTGCTCCT    600
                   ||||||||||||||||||||||||||||||||||||||||||||||||||
PAB1 A60-9     551 ATGCTTTGAATGGTATGCTGTTGAACGGTCAAGAAATTTATGTTGCTCCT    600

PAB1 WT        601 CACTTGTCCAGAAAGGAACGTGACTCTCAATTGGAAGAGACTAAGGCACA    650
                   ||||||||||||||||||||||||||||||||||||||||||||||||||
PAB1 A60-9     601 CACTTGTCCAGAAAGGAACGTGACTCTCAATTGGAAGAGACTAAGGCACA    650

PAB1 WT        651 TTACACTAACCTTTATGTGAAAAACATCAACTCCGAAACTACTGACGAAC    700
                   ||||||||||||||||||||||||||||||||||||||||||||||||||
PAB1 A60-9     651 TTACACTAACCTTTATGTGAAAAACATCAACTCCGAAACTACTGACGAAC    700

PAB1 WT        701 AATTCCAAGAATTGTTTGCCAAATTTGGTCCAATTGTTTCTGCCTCTTTG    750
                   ||||||||||||||||||||||||||||||||||||||||||||||||||
PAB1 A60-9     701 AATTCCAAGAATTGTTTGCCAAATTTGGTCCAATTGTTTCTGCCTCTTTG    750

PAB1 WT        751 GAAAAGGATGCTGATGGAAAATTGAAGGGTTTCGGGTTTGTTAACTACGA    800
                   ||||||||||||||||||||||||||||||||||||||||||||||||||
PAB1 A60-9     751 GAAAAGGATGCTGATGGAAAATTGAAGGGTTTCGGGTTTGTTAACTACGA    800

PAB1 WT        801 AAAGCATGAAGACGCTGTGAAAGCTGTTGAAGCTTTGAATGACTCTGAAC    850
                   ||||||||||||||||||||||||||||||||||||||||||||||||||
PAB1 A60-9     801 AAAGCATGAAGACGCTGTGAAAGCTGTTGAAGCTTTGAATGACTCTGAAC    850

PAB1 WT        851 TAAATGGAGAAAAGTTATACGTTGGTCGTGCCCAAAAGAAGAATGAACGT    900
                   ||||||||||||||||||||||||||||||||||||||||||||||||||
PAB1 A60-9     851 TAAATGGAGAAAAGTTATACGTTGGTCGTGCCCAAAAGAAGAATGAACGT    900

PAB1 WT        901 ATGCATGTCTTGAAGAAGCAATACGAAGCTTACAGATTGGAAAAAAATGGC    950
                   ||||||||||||||||||||||||||||||||||||||||||||||||||
PAB1 A60-9     901 ATGCATGTCTTGAAGAAGCAATACGAAGCTTACAGATTGGAAAAAAATGGC    950
```

```
PAB1 WT          951 CAAGTACCAAGGTGTTAATTTGTTTGTTAAGAACTTAGATGACAGCGTTG   1000
                     |||||||||||||*||||||||||||||||||||||||||||||||||||
PAB1 A60-9       951 CAAGTACCAAGGTCTTAATTTGTTTGTTAAGAACTTAGATGACAGCGTTG   1000

PAB1 WT         1001 ATGACGAAAAGTTGGAAGAAGAATTTGCTCCATATGGTACTATCACTTCT   1050
                     ||||||||||||||||||||||||||||||||||||||||||||||||||
PAB1 A60-9      1001 ATGACGAAAAGTTGGAAGAAGAATTTGCTCCATATGGTACTATCACTTCT   1050

PAB1 WT         1051 GCAAAGGTTATGAGAACCGAAAACGGTAAGTCTAAGGGTTTTGGTTTTGT   1100
                     ||||||||||||||||||||||||||||||||||||||||||||||||||
PAB1 A60-9      1051 GCAAAGGTTATGAGAACCGAAAACGGTAAGTCTAAGGGTTTTGGTTTTGT   1100

PAB1 WT         1101 TTGTTTCTCAACTCCAGAGGAAGCTACTAAGGCCATTACAGAAAAGAACC   1150
                     ||||||||||||||||||||||||||||||||||||||||||||||||||
PAB1 A60-9      1101 TTGTTTCTCAACTCCAGAGGAAGCTACTAAGGCCATTACAGAAAAGAACC   1150

PAB1 WT         1151 AACAAATTGTTGCTGGTAAGCCATTATACGTTGCCATTGCTCAAAGAAAA   1200
                     ||||||||||||||||||||||||||||||||||||||||||||||||||
PAB1 A60-9      1151 AACAAATTGTTGCTGGTAAGCCATTATACGTTGCCATTGCTCAAAGAAAA   1200

PAB1 WT         1201 GACGTAAGACGTTCTCAATTGGCTCAACAAATCCAAGCCAGAAATCAAAT   1250
                     ||||||||||||||||||||||||||||||||||||||||||||||||||
PAB1 A60-9      1201 GACGTAAGACGTTCTCAATTGGCTCAACAAATCCAAGCCAGAAATCAAAT   1250

PAB1 WT         1251 GAGATACCAGCAAGCTACTGCTGCCGCTGCCGCCGCCGCTGCCGGTATGC   1300
                     ||||||||||||||||||||||||||||||||||||||||||||||||||
PAB1 A60-9      1251 GAGATACCAGCAAGCTACTGCTGCCGCTGCCGCCGCCGCTGCCGGTATGC   1300

PAB1 WT         1301 CAGGTCAATTCATGCCTCCAATGTTCTATGGTGTTATGCCACCAAGAGGT   1350
                     ||||||||||||||||||||||||||||||||||||||||||||||||||
PAB1 A60-9      1301 CAGGTCAATTCATGCCTCCAATGTTCTATGGTGTTATGCCACCAAGAGGT   1350

PAB1 WT         1351 GTTCCATTCAACGGTCCAAACCCACAACAAATGAACCCAATGGGCGGTAT   1400
                     ||||||||||||||||||||||||||||||||||||||||||||||||||
PAB1 A60-9      1351 GTTCCATTCAACGGTCCAAACCCACAACAAATGAACCCAATGGGCGGTAT   1400

PAB1 WT         1401 GCCAAAGAACGGCATGCCACCTCAATTTAGAAATGGTCCGGTTTACGGCG   1450
                     ||||||||||||||||||||||||||||||||||||||||||||||||||
PAB1 A60-9      1401 GCCAAAGAACGGCATGCCACCTCAATTTAGAAATGGTCCGGTTTACGGCG   1450

PAB1 WT         1451 TCCCCCCACAAGGTGGTTTCCCAAGAAATGCCAACGATAACAACCAATTT   1500
                     |||||||||||||||||||||||||*||||||||||||||||||||||||
PAB1 A60-9      1451 TCCCCCCACAAGGTGGTTTCCCAAGTAATGCCAACGATAACAACCAATTT   1500

PAB1 WT         1501 TATCAACAAAAGCAAAGACAAGCTTTGGGTGAACAATTATACAAGAAGGT   1550
                     ||||||||||||||||||||||||||||||||||||||||*|||||||||
PAB1 A60-9      1501 TATCAACAAAAGCAAAGACAAGCTTTGGGTGAACAATTATTCAAGAAGGT   1550

PAB1 WT         1551 TTCTGCTAAGACTTCAAATGAAGAAGCAGCTGGTAAAATTACTGGTATGA   1600
                     ||||||||||||||||||||||||||||||||||||||||||||||||||
PAB1 A60-9      1551 TTCTGCTAAGACTTCAAATGAAGAAGCAGCTGGTAAAATTACTGGTATGA   1600

PAB1 WT         1601 TTTTGGATTTGCCACCTCAAGAGGTCTTCCCATTGTTGGAAAGTGATGAA   1650
                     |||||||||||||||||||||*||||||||||||||||||||||||||||
PAB1 A60-9      1601 TTTTGGATTTGCCACCTCAAGAAGTCTTCCCATTGTTGGAAAGTGATGAA   1650

PAB1 WT         1651 TTGTTCGAACAACACTACAAAGAAGCTTCTGCTGCCTATGAGTCTTTCAA   1700
                     ||||||||||||||||||||||||||||||||||||||||||||||||||
PAB1 A60-9      1651 TTGTTCGAACAACACTACAAAGAAGCTTCTGCTGCCTATGAGTCTTTCAA   1700

PAB1 WT         1701 AAAGGAGCAAGAACAACAAACTGAGCAAGCTTAA   1734
                     ||||||||||||||||||||||||||||||||||
PAB1 A60-9      1701 AAAGGAGCAAGAACAACAAACTGAGCAAGCTTAA   1734
```

Supplementary Figure S5. Sequence alignment of *PAB1* wild type and *PAB1 A60-9* coding sequences. Missense and silent mutations are pointed out with red or black star symbols, respectively.

Supplementary Table S1. List of yeast strains constructed and used in this study.

**Strain**	**Genotype**	**Source**
BY4741 –HULM	*MATa*; *his3Δ1; leu2Δ0; met15Δ0; ura3Δ0*	Euroscarf
BY4741c	*MATa; his3Δ1; leu2Δ0; met15Δ0; ura3Δ0 [pYX012; pYX022; pYX052; YCplac33]*	This study
BY4741 Pab1(+)	*MATa; his3Δ1; leu2Δ0; met15Δ0; ura3Δ0 [pYX012; pYX022; pYX052; YCplac33PAB1]*	This study
BY4741 Pab1GFP	*MATa; his3Δ1; leu2Δ0; met15Δ0; ura3Δ0 [pYX012; pYX022; pYX052; YCplac33PAB1GFP]*	This study
BY4741 –HLM	*MATa; his3Δ1; leu2Δ0; met15Δ0; ura3Δ0 [YCplac33]*	This study
BY4741 -HLM Pab1(+)	*MATa; his3Δ1; leu2Δ0; met15Δ0; ura3Δ0 [YCplac33PAB1]*	This study
BY4741 -HLM Pab1A60-9	*MATa; his3Δ1; leu2Δ0; met15Δ0; ura3Δ0 [YCplac33A60-9]*	This study
BY4741 -HLM *pan2Δ*	*MATa; his3Δ1; leu2Δ0; met15Δ0; ura3Δ0; YGL094c::kanMX4 [YCplac33]*	This study
BY4741 -HLM *pan3Δ*	*MATa; his3Δ1; leu2Δ0; met15Δ0; ura3Δ0; YKL025c::kanMX4 [YCplac33]*	This study
CEN.PK113-5D	*MATa; MAL2-8c; SUC2; ura3-52*	1
CEN.PK113-5Dc	*MATa; MAL2-8c; SUC2; ura3-52 [YCplac33]*	This study
CEN.PK113-5D Pab1(+)	*MATa; MAL2-8c; SUC2; ura3-52 [YCplac33PAB1]*	This study
CEN.PK113-5D Pab1GFP	*MATa; MAL2-8c; SUC2; ura3-52 [YCplac33PAB1GFP]*	This study
CEN.PK113-5D Pab1A60-9	*MATa; MAL2-8c; SUC2; ura3-52 [YCplac33PAB1A60-9]*	This study
CEN.PK113-5D PAB1-GFP	*MATa; MAL2-8c; SUC2; ura3-5; PAB1-GFP::His3MX6 [YCplac33]*	This study
CEN.PK113-5D Pab1(+)-GFP	*MATa; MAL2-8c; SUC2; ura3-5; PAB1-GFP::His3MX6 [YCplac33PAB1GFP]*	This study
CEN.PK113-5D Pab1Y514F	*MATa; MAL2-8c; SUC2; ura3-52 [YCplac33PAB1*^{Y514F}*]*	This study
CEN.PK113-5D PAB1-GFP Pab1A60-9	*MATa; MAL2-8c; SUC2; ura3-52; PAB1-GFP::His3MX6 [YCplac33PAB1A60-9]*	This study
CEN.PK113-5D PAB1-GFP Pab1Y514F	*MATa; MAL2-8c; SUC2; ura3-52; PAB1-GFP::His3MX6 [YCplac33PAB1*^{Y514F}*]*	This study
CEN.PK113-11C	*MATa; MAL2-8c; SUC2; ura3-52; his3Δ1*	1
CEN.PK113-11C PAB1-GFP EDC3-mCh	*MATa; MAL2-8c; SUC2; ura3-52; his3Δ1; YEL015W::hphMX4; PAB1-GFP::His3MX6 [pRP1574]*	This study
CEN.PK113-11C PAB1(+)-GFP EDC3-mCh	*MATa; MAL2-8c; SUC2; ura3-52; his3Δ1; YEL015W::hphMX4; PAB1-GFP::His3MX6 [pRP1657]*	This study

Supplementary Table S2. List of oligonucleotides used in this study. When present, restriction sites are underlined.

**Oligonucleotide**	**Sequence**
Pab1 PstI fw	5’-GAT TTA CTG CAG GTA TAT ATA TTT GCG TGT AAG TGT GTG T-3’
Pab1 SalI rev	5’-ATT ATA GTC GAC TAG AGC ATT AAG CTT GCT CAG TTT GT-3’
ΔEdc3 fw	5’-ATG TCA CAA TTT GTT GGT TTC GGA GTA CAA GTG GAG CTA AAA GAT CGC CAG ATC TGT TTA GCT TG-3’
ΔEdc3 rev	5’-TTA CAA ATC TAA TAG CAG GGA CCC GTC AGT GAC GAA AAG ATC ACA GAG CTC GTT TTC GAC ACT GG-3’
Edc3 fw	5’-GAT GTT CGC AGT TTT CAT ATT TGT GAG A-3’
Edc3 rev	5’-AGA AAA CAT AGT TAG CTC GAA TAG TTT AGT A-3’
Hph fw	5’-ATA TGA AAA AGC CTG AAC TCA CCG AC-3’
Hph rev	5’-TCT ATT CCT TTG CCC TCG GAC-3’
Pab1 GFP fw	5’-AGT CTT TCA AAA AGG AGC AAG AAC AAC AAA CTG AGC AAG CTC GGA TCC CCG GGT TAA TTA A-3’
Pab1 GFP rev	5’-GTT TGT TGA GTA GGG AAG TAG GTG ATT ACA TAG AGC ATT AGA ATT CGA GCT CGT TTA AAC TGG-3’
Pab1 GFP SalI rev	5’-ATT ATA GTC GAC TGG CGC GCC TTA TTT GTA TAG TT-3’
Pab1 mut fw	5’-AAG CTT GCA TGC CTG CAG GTA TAT A-3’
Pab1 mut rev	5’-GGG TCG ACT AGA GCA TTA AGC TT-3’
Pgk fw	5’-GTG TTG CTT TCT TAT CCG AAA AG-3’
Pgk rev	5’-TGA ACC CGA ACA TAG AAA TAT CG-3’
Pab1 Y514F fw	5’-CAC CTC AAG AAG TCT TCC CAT TGT TGG AAA GT-3’
Pab1 Y514F rev	5’-CAA TGG GAA GAC TTC TTG AGG TGG CAA ATC-3’
TPIp PstI fw	5’-GAT TTA CTG CAG CCT GAC GTC TAA GAA ACC ATT A-3’
TPIp EcoRI rev	5’-CTT AGA GAA TTC TCT TCG CTA TTA CGC CAG CT-3’

SUPPLEMENTARY REFERENCES

1 Entian, K. & Kötter, P. in *Yeast gene analysis* Vol. 26 (eds JPA Brown & MF Tuite) 431-449 (San Diego California Academic Press, 1998).
